# A systematic review of qualitative research on HIV and food insecurity in high-income countries

**DOI:** 10.1097/QAD.0000000000004552

**Published:** 2026-05-25

**Authors:** Emily Jay Nicholls, P Bicarregui, Anna Brewster, Indrajit Ghosh, Stuart Flanagan, Shema Tariq

**Affiliations:** a University College London; b University Hospitals Sussex NHS Foundation Trust; c The Food Chain; d University College London Hospitals NHS Foundation Trust; e Central and North West London NHS Foundation Trust, UK.

**Keywords:** financial hardship, food insecurity, HIV, qualitative, systematic review

## Abstract

**Objective(s):**

Food insecurity leads to adverse health outcomes in people living with HIV, including obesity, poor mental health and viral non-suppression. Qualitative studies in low- and middle-income countries have described the impact of food insecurity on medication adherence. However, limited qualitative research exists on the lived experiences of food insecurity among people living with HIV in high-income countries (HICs). We aimed to synthesize qualitative literature on food insecurity among people living with HIV in HICs.

**Design:**

Systematic review and textual narrative synthesis.

**Methods:**

We searched MEDLINE, CINAHL, Scopus, Embase, and PsycINFO, extracting data from included articles on a standardized form in Covidence. We synthesized literature using a textual narrative approach. We conducted quality appraisal (Critical Appraisal Skills Programme Qualitative Studies Checklist) and graded certainty of findings (GRADE-CERQual).

**Results:**

We reviewed 2772 articles, reduced to 940 after deduplication, with 12 articles included after full text screening. Included articles reported on studies conducted in the United States (*n* = 7), and Canada (*n* = 5). We identified three key themes, each with their own sub-themes: the role of structural inequalities in shaping and navigating food insecurity; the impacts of food insecurity on health and wellbeing; and the labour of acquiring of food.

**Conclusions:**

Our review highlights the syndemic nature of food insecurity and HIV; intersecting experiences of multiple structural hardships constellate and interact synergistically to amplify poor health outcomes. Interventions should address the multiple and reinforcing social and structural conditions that shape food insecurity among people living with HIV.

## Introduction

Food insecurity is defined as having “limited access to food, at the level of individuals or households, due to lack of money or other resources” [1]. It also encompasses situations in which “the availability of nutritionally adequate and safe foods or the ability to acquire acceptable foods in socially acceptable ways is limited or uncertain” [2]. It is associated with a wide range of health impacts including diabetes [3], hyperlipidaemia [4], asthma [5], depression and anxiety [6].

There is a high prevalence of material deprivation among people living with HIV, with a bidirectional relationship [7]. Food insecurity and HIV are syndemic, interacting synergistically to amplify negative health outcomes [8,9]. These interactions are further shaped by other social determinants of health, such as poverty, housing insecurity, stigma and structural inequalities. Systematic reviews of quantitative studies in diverse settings have identified multidimensional impacts of food insecurity on people living with HIV including poor mental health [10,11], reduced adherence to antiretroviral therapy (ART) [12], and viral non-suppression [13].

Food insecurity among people living with HIV has been well documented in low- and middle-income countries (LMICs), however it is also a significant issue in high-income countries (HICs), with prevalence estimates in the United States and Canada ranging from 36% to 71% [14,15]. It is more likely to affect households with children, those in large urban areas, and with low incomes [16] and has been associated with being a current smoker [16] and non-medicinal drug use [16,17]. Most quantitative research in HICs has been conducted with women, and among this population, has linked food insecurity with obesity [18], higher viral load [19], lower CD4^+^ cell count [19], elevated inflammation [20] poorer cognitive performance [21] and increased levels of HIV-related stigma [22].

Most qualitative studies on food insecurity in people living with HIV have been undertaken in LMICs [23–26]. There have been few such studies in HICs, meaning we lack data on the structural and contextual factors specific to these settings. Differences in welfare provision, healthcare systems and social supports mean that the experiences and consequences of food insecurity are likely to differ from those documented in LMICs. Context-specific data are important to understand the mechanisms by which food insecurity shapes clinical outcomes, as well as informing intervention development [27]. Such insights can help HIV care providers recognize and respond to the ways food insecurity shapes treatment engagement and broader experiences of health and wellbeing.

We present a systematic review of qualitative studies on food insecurity among people living with HIV in HICs.

## Methods

We conducted a systematic review, adhering to the Preferred Reporting Items for Systematic Reviews and Meta-Analyses (PRISMA) guidelines [28]. This method was selected as we sought to synthesize and critically appraise available evidence and to inform policy and practice [29]. The review protocol was registered on PROSPERO [30].

### Search strategy and inclusion criteria

One author (P.B.) conducted a systematic search of databases (Medline, CINAHL, Scopus, Embase, PsychINFO) on 24^th^ April 2024, having consulted a librarian. The search was updated on 17^th^ September 2025 (EJN) (see supplementary material for search strategy) with no further eligible articles identified.

We included articles reporting qualitative studies in HICs (World Bank definition [31]) published between 2000 and the date of the search, and:

(1) Reporting on studies investigating food insecurity and HIV and/or
(2) Including people living with HIV identified as experiencing food insecurity

We included articles reporting mixed-methods studies, where sufficient qualitative data were presented, and those in which the study population included *both* people with and without HIV (only considering findings pertaining to people living with HIV). We did not apply an *a priori* definition of food insecurity; relying instead on the categorization employed by the original authors. To be eligible, articles needed to be written in English-language, peer-reviewed and reporting on people aged ≥18 years. Grey literature and review articles were excluded.

### Data extraction and management

Two authors (E.J.N., P.B.) independently extracted data from all included articles using a standardized form in Covidence [32], capturing bibliographic information, methods, setting, recruitment, involvement of people with lived experience, themes and key findings.

### Qualitative synthesis

We conducted a textual narrative synthesis of included studies [33]. Extracted data from individual studies were exported from Covidence to Excel. E.J.N. reviewed extracted data from individual studies, synthesizing findings by organizing study-level themes into broader analytic themes. Informed by syndemic theory [8], we paid particular attention to the ways concurrent experiences of hardship interacted.

### Quality appraisal

E.J.N. and P.B. completed the Critical Appraisal Skills Programme (CASP) Qualitative Studies Checklist [34] during data extraction. ST and EJN used this as a basis to assess quality as ‘high’, ‘moderate’ or ‘low’. We also considered whether people with lived experience of HIV and food insecurity and/or community organisations had been included in the design, conduct or interpretation.

### Assessment of confidence in findings

E.J.N. and S.T. determined confidence in findings using GRADE-CERQual [35]. This involved assessing, for each sub-theme, the methodological limitations (using CASP appraisal results), coherence (coherence of evidence across studies), adequacy (number of included studies and adequacy of their data), relevance (the extent to which this finding was specific to our population).

## Results

### Characteristics of included articles

**Fig. 1:**
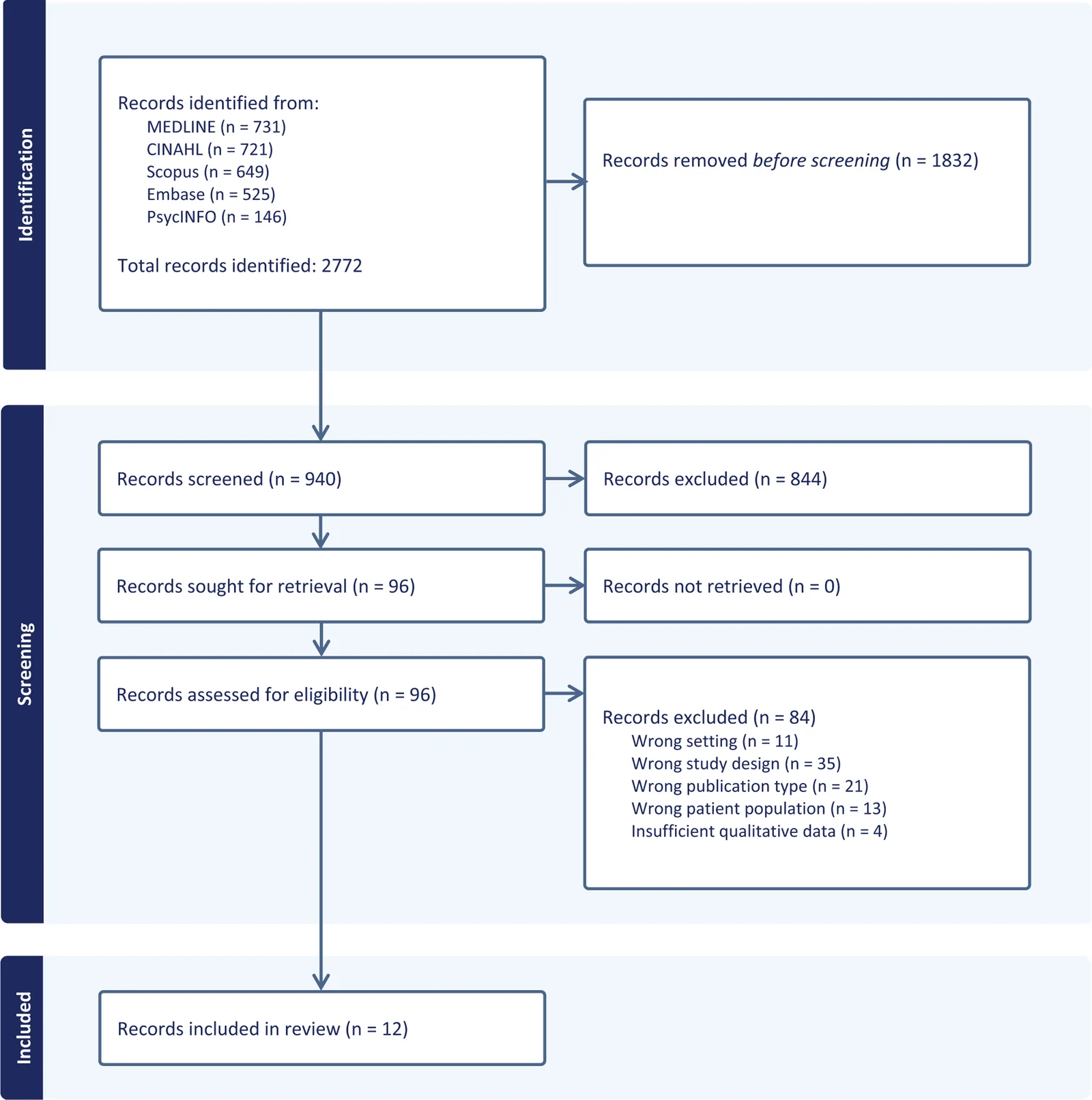
PRISMA flow chart.

We reviewed 2772 articles, reduced to 940 following de-duplication. After initial title and abstract screening, 96 underwent full text review, with 84 articles excluded (see Fig. 1).

All 12 included articles reported on studies conducted in North America (USA *n* = 7; Canada *n* = 5). Three reported on studies recruiting from the United States not-for-profit organization Project Open Hand [36–38]. Two were based on analyses of data from the U.S.-based Women's Interagency HIV Study [now part of the Multicenter AIDS Cohort Study-WIHS Combined Cohort Study (MWCCS)] [39,40]. Articles were published between 2010 and 2023, with seven published in 2020 or later; only one discussed COVID-19.

Almost all included articles reported on interview studies (*n* = 11); one reported on community-based participatory workshops. At least 50% of participants in included articles were from racially minoritized communities. Three studies focussed exclusively on the experiences of women [39–41], one including transgender women [41].

### Quality assessment and GRADE-CERQual

Included articles were of an overall high quality, with 10 graded as ‘high’, one ‘moderate’ and one ‘low’ (Table 1).

**Table 1:** Summary of included studies.

Lead author	Title	Year of publication	Country	Name of study/intervention	Aim of study	Theoretical framework	Study design	Participant population	Number of participants	Participant demographics^a^	Recruitment setting	Inclusion of people living with HIV/community	Summary of findings	Quality appraisal
Miewald	Negotiating the Local Food Environment: The Lived Experience of Food Access for Low-Income People Living with HIV/AIDS	2010	Canada	Good Eats workshop	Explore how HIV, poverty, gender and addiction shape the strategies people living with HIV on low incomes use to access food.		Participatory workshops	People living with HIV	10	• 4 cis women, 2 trans women, 4 cis men. • Ages 20's-early 50's, most participants in their 40's. • Majority of participants were Indigenous. • Over half of participants had been involved in sex work • 9 participants were either currently or had previously been using drugs. No table of demographics included.	Community organization and social services agencies	Participatory methods used, but it is unclear whether people with lived experience have been included in the design, conduct or interpretation.	Navigating multiple food provision services required time, effort and knowledge. Drug use, long queues for accessing services and concerns that attending services might be perceived as a surrogate marker for living with HIV were barriers for accessing food.	Low
Whittle	How food insecurity contributes to poor HIV health outcomes: Qualitative evidence from the San Francisco Bay Area	2016	USA	Project Open Hand Food=Medicine Program	To understand the impact of food insecurity on HIV-related health behaviours	Socio-ecological model	Interviews	People living with HIV, over 18 years, Spanish or English speaking, low income, history of good adherence to Project Open Hand services	34	• 82% Male, 18% Female • 50% White, 47% Black/African American, 18% Hispanic, 15% Other^b^	Voluntary sector service provider	Not apparent, although the primary interviewer was volunteering within the service	Food insecurity can have a negative impact on HIV medication adherence and clinic attendance as well as significant effects on mental health and wellbeing.	High
Whittle	Experiences with food insecurity and risky sex amongst low-income people living with HIV/AIDS in a resource-rich setting	2015	USA	Project Open Hand Food=Medicine Program	Investigate the relationship between food insecurity and sexual risk behaviours.		Interviews	People living with HIV, over 18 years, Spanish or English speaking, low income, history of good adherence to Project Open Hand services	34	• 82% Male, 18% Female • 50% White, 47% Black/African American, 18% Hispanic, 15% Other^b^	Voluntary sector service provider	No	Food insecurity can contribute to transactional sex, including influencing ability to advocate for condom use.	High
Whittle	Food insecurity, chronic illness, and gentrification in the San Francisco Bay Area: An example of structural violence in United States public policy	2015	USA	Project Open Hand	To investigate how food insecurity manifests among particular populations; the structural determinants and the mechanisms by which they are translated in to food insecurity; and the impact on health outcomes among people living with HIV on low-incomes	Structural violence	Interviews	People living with HIV enrolled in Project Open Hand. Over 18, low income, English or Spanish speaking, living with HIV, history of good adherence to Project Open Hand services	34	• 82% Male, 18% Female • 50% White, 47% Black/African American, 18% Hispanic, 15% Other^b^	Voluntary sector service provider	No	Participants experienced insufficient quality and quantity of food, driven by high rents and low income.	High
Whittle	Precarity and health: Theorizing the intersection of multiple material-need insecurities, stigma, and illness among women in the United States	2020	USA	Women's Interagency HIV Study	To understand material-need insecurities in addition to food insecurity, how they intersect and their health impacts		Interviews	Women over 50 years, living with HIV and classified as food insecure using the Household Food Security Survey Module	26 women living with HIV (38 participants total)	• All participants over 50 • 66% African American 13% Hispanic 16% White 5% Other • 68% living with HIV 32% HIV negative^a^	Cohort study	No	The financial prioritisation of accessing healthcare, bills and rent over food quantity and quality leads to worsening of physical and mental health.	High
Miewald	“I do my best to eat while I’m using”: Mapping the foodscapes of people living with HIV/AIDS who use drugs	2019	Canada	Food as Harm Reduction	Explore the lived experience and spaces of food access among people living with HIV who use drugs		Mixed methods (survey and interviews)	Living with HIV and have used non-prescribed drugs (not including alcohol or cannabis) in 30 days prior to participating in survey	20 interviews (60 participants in total – survey respondents not reported in this review)	• 90% Male • 50% Caucasian, 45% Indigenous, 5% Other ethnicity	Community organisation	Yes: people who are living with HIV and use drugs on community advisory board and peer researchers. Peer researchers were involved in research design, administration, interpretation and dissemination	Food assistance support was part of the daily routines of people living with HIV who use drugs participating in this study. These are considered “spaces of care” where participants can access nutrition as well as social support and connection.	High
Sernick	In the midst of plenty: Experiences of food insecurity amongst women living with HIV in Vancouver, Canada	2022	Canada	Sexual Health and HIV/AIDS: Women's Longitudinal Needs Assessment (SHAWNA)	To explore the relationships between sociocultural and structural factors, individual behaviour and environment in relation to food insecurity among women living with HIV	Socio-ecological framework	Interviews	Women (cis and transgender/gender diverse) living with HIV	64	• 54 cis women, 10 people assigned male at birth (3 identified as transgender, 3 as two spirit, 2 as women, 1 as heterosexual female, 1 as gender queer) • 48% Indigenous, 14% White, 9% Black African, 5% Mixed ethnicity or other visible minorities	Peer outreach and SHAWNA cohort study	SHAWNA initiated through community consultation. Interview guide developed with women living with HIV and community partners. Women living with HIV, sex workers, Indigenous, African, migrant and transwomen involved as part of the SHAWNA research team.	Women living with HIV may experience structural and sociocultural barriers to food and food access. This can include limited access to culturally specific foods and barriers relating to gender and safety.	High
Leddy	Intersections of food insecurity, violence, poor mental health and substance use among US women living with and at risk for HIV: Evidence of a syndemic in need of attention	2021	USA	Women's Interagency HIV Study (WIHS), now part of the Multicenter AIDS Cohort Study-WIHS Combined Cohort Study (MWCCS)	To explore the intersections of food insecurity and intimate partner violence and how they influence HIV risk and treatment behaviour among women living with and at increased risk of HIV	Syndemics	Interviews	Women who reported physical, sexual or emotional violence in the past year at their most recent visit; marginal, low or very low food security at or before their most recent visit	12 people living with HIV (24 total: respondents who were not living with HIV not reported in this study)	Of whole sample (including participants who were not living with HIV): 16 Black/African American, 4 White, 4 Other ethnicity	WIHS sites	No	Intersecting food insecurity, intimate partner violence, drug use and poor mental health undermine ART adherence and clinic attendance.	High
Chayama	How food support improves mental health among people living with HIV: A qualitative study	2023	USA	Project Open Hand (POH) Food=Medicine Program	To explore the mental health of people living with HIV who are receiving medically appropriate food support, and to understand how interventions like this might impact mental health		Interviews	People living with HIV participating in Food=Medicine Program. English or Spanish speaking, low income, over 18 years	34	• 85% Male, 15% Female • 44% White, 41% Black/African American, 3% Asian/Pacific Islander, 21% Hispanic/Latino, 15% Native American	Voluntary sector service provider	No	Participating in this food assistance programme improved the mental health of participants.	High
Odhiambo	Structural violence and the uncertainty of viral undetectability for African, Caribbean and Black people living with HIV in Canada: an institutional ethnography	2023	Canada		To explore the impact of structural violence on retention in HIV care and treatment adherence	Structural violence	Interviews	Living with HIV and self-identifying as African, Caribbean or Black ethnicity	20 people living with HIV (plus 15 healthcare workers not reported in this review)	All participants of African, Caribbean or Black ethnicities	Community based organizations and healthcare centres	No	Food insecurity amongst people living with HIV of African, Caribbean and Black ethnic backgrounds had a detrimental impact on ART adherence. This was linked with experiences of housing insecurity.	High
Joy	Disciplined appetites: Reimagining food and nutrition programs for people living with HIV and AIDS	2023	Canada		To explore beliefs, values and experiences of food and nutrition support services among people living with HIV to inform the design and implementation of future programmes	Critical health geography and critical dietetics	Interviews	Living with HIV, over 19 years, participants didn’t have to be experiencing food insecurity	12	• 4 Black, 6 White. 2 Black/Asian • 8 Men. 4 women • 8 Heterosexual, 4 Gay	Social media, HIV service organisations, community partners, professional networks of the principal investigator	No	Financial situation and HIV stigma are key barriers to accessing food and nutrition programmes	Moderate
Bleasdale	Socio-Structural Factors and HIV Care Engagement among People Living with HIV during the COVID-19 Pandemic: A Qualitative Study in the United States	2022	USA		To understand the ways socio-cultural factors influenced care engagement during the COVID-19 pandemic		Interviews	People living with HIV and receiving or had received food assistance in last 30 days	25	• 52% Male. 48% Female 68% Black/Africa American, 28% White, 4% Other ethnicity, 8% Hispanic/Latio/a	Sexual health clinics, LGBTQ+ community centre, health service organisation for LGBTQ+ people	No, but health workers and researchers reviewed interview guides	ART adherence and clinic attendance were negatively impacted by food insecurity during the COVID-19 pandemic.	High

a Only the experiences of people living with HIV are included in this review.

b Participants could self-identify as multiple categories.

Three themes were graded as high confidence in the GRADE-CERQual assessment (Table 2). Firstly, the deleterious impact of multiple stigmas, including HIV stigma and the stigma associated with multiple material deprivation. Secondly, food insecurity negatively impacts ART adherence and clinic attendance. Finally, that food insecurity has a negative impact on the mental health of people living with HIV.

**Table 2:** GRADE CERQual summary.

Summary of review finding	Studies contributing to the review finding	Assessment of methodological limitations	Assessment of coherence	Assessment of adequacy	Assessment of relevance	Overall CERQual assessment of confidence in the evidence	Explanation of CERQual assessment
*The role of structural inequalities in shaping and navigating food insecurity*
Gender	[39]	No concerns	No concerns	Serious concerns: only one paper	Serious concerns: not specific to people living with HIV	Low	Finding based on only one study and not specific to people living with HIV.
Ethnicity	[41,42]	No concerns	Minor concerns: different focus of studies impacts coherence	Serious concerns: only two papers	Moderate concerns: some specificity to people living with HIV with some elements of finding, but not all	Low	Finding based on only two studies, with slightly different focus, impacting coherence. Additionally, while there is some specificity to people living with HIV, this is only in some aspects of the finding and not all.
Drug use	[39,41,43,44]	No concerns [39,41,43] Serious concerns [44]	No concerns: overall coherence across studies	No concerns: four studies contributing to this finding	Minor concerns: some lack of certainty how specific this finding is to the experiences of people living with HIV	Moderate	Based on four studies of a generally high quality. Limited discussion of intersection between HIV, drug use and food insecurity
Multiple stigmas	[40,41,44,45,46]	No concerns: [40,41,45] Moderate: [46] Serious: [44]	No concerns: overall coherence across studies	No concerns: five studies contributing to this finding	No concerns: finding specific to HIV	High	Based on five studies, three of high quality. Coherence across studies and finding highly relevant to people living with HIV
Intersectional material disadvantage	[37,40,41,42,44,46,47]	No concerns: [37,40,41,42,47] Moderate concerns: [46] Serious concerns: [44]	No concerns: overall coherence across studies	No concerns: seven studies contributing to this finding	Serious concerns: not specific to people living with HIV	Moderate	Based on seven studies, the majority of which are of a high quality. Coherence across studies, however, these findings are not specific to people living with HIV.
*The impacts of food insecurity among people living with HIV*
Engagement with HIV care	[39,42,45,47]	No concerns	No concerns: overall coherence across studies	No concerns: four studies contributing to this finding	No concerns: highly specific to people living with HIV	High	Based on four studies, all of a high quality. There is coherence across studies and this finding is highly specific to people living with HIV.
Mental health	[38,39,45,47]	No concerns	No concerns: overall coherence across studies	No concerns: five studies contributing to this finding	Minor concerns: some elements of finding specific to people living with HIV, whereas other elements likely to be broadly applicable to food insecurity	High	Based on four high quality studies with overall coherence. Some elements of finding may be broadly applicable to food insecurity, however there is some specificity to people living with HIV
*The pursuit of food*
Strategies for acquiring food	[36,37,39,43]	No concerns	No concerns: overall coherence across studies	No concerns: four studies contributing to this finding	Major concerns: not specific to people living with HIV	Moderate	Based on four high quality studies with overall coherence. Not specific to people living with HIV
Experiences of food support services and programmes	[37,38,41,43,44,46]	No concerns: [37,38,41,43] Moderate concerns: [46] Serious concerns: [44]	No concerns: this finding captures both positive and negative experiences of food support services. However, there is an overall coherence across studies.	No concerns: six studies contributing to this finding	Major concerns: not specific to people living with HIV	Moderate	Based on six studies, the majority of which are of a high quality. There is overall coherence across studies, however this finding is not specific to people living with HIV.
Barriers to accessing food support services and programmes	[36–38,40,41,43–46]	No concerns: [36–38,40,41,43,45] a Moderate concerns: [46] Major concerns [44]	No concerns: overall coherence across studies	No concerns: nine studies contributing to this finding	Moderate concerns: while some elements of this finding are specific to people with HIV, many aspects are not	Moderate	Based on nine studies, the majority of which are of a high quality. Overall coherence across studies, however while some elements of this finding are specific to people living with HIV, many aspects are not.

We identified three overarching themes, with sub-themes. These are presented below, with illustrative quotes for each sub-theme (Table 3).

**Table 3:** Illustrative quotes.

Theme	Quote
The role of structural inequalities in shaping and navigating food insecurity
Gender	“Women who depended on intimate partners to meet their material needs also described feeling unable to leave abusive relationships, particularly if they wanted to ensure consistent access to resources for their children” (p. 7) [39]
Ethnicity	“For Indigenous WLWH or participants who had im/migration backgrounds, culturally specific foods formed an important part of food security, as those accustomed to preparing and eating culturally specific foods struggled to maintain the same nutritional integrity in their diets given the available food options.” (p. 6) [41]
Drug use	“[S]ome [food providers] do not allow anyone under the influence of drugs or alcohol to be served and individuals can be banned if they continually break agency rules. Many members of the Good Eats group were critical of policies that excluded people based on drug use” (p. 517–518) [44] “[D]rug use was found to decrease appetite and increase paranoia, which presented barriers to food access for some. Feeling stigmatized for using drugs in some settings also means that individuals may not use the food services available to them.” (p. 101) [43]
Multiple stigmas	“[P]articipants articulated worries about stigma when attending these institutions. These fears concerned both revealing one's dependence on charity for food and inadvertently disclosing one's HIV status by taking ART medications with the meals served.” (p. 232) [45]
Intersectional material disadvantage	“Women usually described working (sometimes multiple) part-time jobs, often on informal or atypical contractual arrangements (e.g. ad hoc shifts, cash payment), sometimes during odd hours (e.g. night shifts) or past retirement age against their wishes, and mostly without access to workplace benefits including health insurance” (p. 4) [40] “Black people could not access or afford healthier and culturally responsive food options necessary to maintain a healthy lifestyle and accommodate their health conditions, dietary restrictions and ART prescription requirement because of inadequate income and competing priorities.” (p. 8) [42]
The impacts of food insecurity on people living with HIV
Engagement with HIV care	“As with ART adherence, it also emerged that food insecurity could undermine engagement in care in subtler ways. Again, these were embedded in the day-to-day lived experience of food insecurity and destitution, and included missing appointments because of preoccupation with food struggles” (p. 232) [45]
Mental health	“Participants frequently evoked the sense of stress and anxiety inherent to food insecurity that is captured in its definition, and, moreover, described how experiences with food insecurity directly contributed to periods of depression.” (p. 233) [45]
The pursuit of food
Strategies for acquiring food	“Faced with these issues, participants turned to a multitude of strategies aimed at procuring either food directly or money to buy food, many of which they found personally uncomfortable and some of which would widely be considered socially unacceptable. These included long-term dependence on friends, family, and charity (mainly soup kitchens and food banks and pantries), as well as risky strategies such as sexual exchange, stealing food, and selling controlled substances” (p. 159) [37]
Experiences of food support services and programmes	“Participants spoke of doing their “rounds” to various food providers, depending upon the day and time. For example, one participant who was homeless at the time did what he called “the Hastings Shuffle” in reference to the main street in the DTES where many food providers are located. He started with breakfast at a church soup kitchen and then came to LifeSkills for lunch. For dinner it was another social service agency or another church.” (p. 516) [44]
Barriers to accessing food support services and programmes	“Some WLWH were not able to travel to food services due to the cost of transportation, family responsibilities, as well as mobility limitations, including medication side effects such as illness and fatigue, which impacted women's ability to stand in line at food services” (p. 6) [41]

### The role of structural inequalities in shaping and navigating food insecurity

#### Gender

Gender shaped vulnerability to and experiences of food insecurity. In one study, women living with HIV experiencing intimate partner violence described how a reliance on partners for material needs could be a barrier to leaving abusive relationships [39]. The combined effects of food insecurity and violence led to feelings of low self-worth [39]. There were gender-specific considerations in access to food support services, described later.

#### Ethnicity

One study reporting on the experiences of people of Black ethnicities in Canada (who were not recruited on the basis of experiencing food insecurity), highlighted the impact of multiple and concurrent material deprivations in this population, particularly in relation to ART adherence [42]. Lack of affordable healthy food compounded this, and left participants unable to maintain diets needed to manage other health conditions such as diabetes and hypertension [42]. Furthermore, a lack of affordable culturally specific foods [41,42] was a barrier to sustaining a nutritionally adequate diet, particularly in the context of limited storage and preparation facilities [41].

#### Drug use

One article focussed specifically on the experiences of people living with HIV who used drugs [43]; three included participants who discussed drug use [39,41,44]. Drug use was a significant barrier to accessing food support, especially when access was prohibited when intoxicated [44], or due to the stigma of drug use causing discomfort [43].

Drug use shaped experiences of food insecurity whereby procuring drugs was prioritised over food, or food avoided to prolong highs [43]. In one study, women living with HIV described drug use as a coping mechanism for the corrosive effects of violence and food insecurity [39]. Furthermore, drug use often decreased food intake [44] due to appetite suppression [43], disturbance of taste/sensation [43], the practical difficulties of accessing food support services when under the influence of drugs (including symptoms of paranoia)[43]. It also affected quality of food intake, with some participants opting for sweets and “junk” food [44]. However, these experiences were not universal; some participants reported actively trying to ensure sufficient food intake, motivated by a desire to optimize health as someone living with HIV and/or other comorbid conditions [43].

#### Multiple stigmas

Experiences of HIV-related stigma and concerns about being identified as a person living with HIV inhibited access to HIV-specific services [44], or impacted adherence as participants did not want to be seen taking ART when accessing non-HIV specific services [45].

People living with HIV encountered multiple stigmas beyond HIV, including stigma associated with material deprivation such as housing insecurity and/or accessing food assistance [40,46]. In one study, participants described feeling stigmatized by healthcare providers offering dietary advice but not acknowledging structural barriers to maintaining a healthy diet [41]. Internalized stigma of being unable to afford recommended food prevented people from participating in “eating well” programmes [46].

#### Intersectional material disadvantage

Food insecurity did not operate in isolation; rather it was situated within a wider web of interrelated material disadvantage. Participants experienced multiple forms of insecurity [40] and managed numerous competing material needs [42]. Participants went without electricity, heating as well as food [40] or had to prioritize rent, transport [42], household bills or medication [40] over food. Participants were often knowledgeable about healthy diets but unable to afford good quality, healthy food [37,41,46,41].

In a study including women living with HIV aged over 50, participants relied on disability benefits and/or low-paid and insecure jobs [40]. For those in work, this included multiple part time jobs, sometimes at unsociable hours [40]. Remaining in work was particularly challenging for those experiencing chronic or episodic health conditions [40,46]. In one study, job insecurity had increased since the COVID-19 pandemic [47].

State benefits were often difficult to access and maintain [40]; characterized by complex and laborious processes [40]. Benefits rarely covered increasing costs of living [41,42,37] and were subject to sudden withdrawal, especially if individuals exceeded the income threshold (sometimes inadvertently)[40].

Housing insecurity and poor-quality housing often co-existed with food insecurity [42,40,46,47]. High rents were a major cause of financial difficulties [37], especially for those reliant on state benefits [37]. While food was often prioritized over other material needs, shelter was almost always prioritized over food [37], with people choosing lower quality housing to accommodate other competing demands [40].

Those living in poor quality housing were more likely to have limited or unsafe facilities to store or prepare food, including vermin and pest infestations [44,41]. In shelters choice of meals were limited and often unhealthy, with a lack of facilities to or restrictions on food preparation [42].

### The impacts of food insecurity on people living with HIV.

#### Engagement with HIV care

Food insecurity constrained adherence to ART [42,45,47]. For example, individuals missed doses if they were to be taken with food [42]; when they did take antiretrovirals in the absence of food, they were conscious of increased side effects [45]. Prolonged duration of food insecurity negatively impacted adherence, partly due to persistent gastrointestinal side-effects [45].

Lived experience of food insecurity impacted negatively on motivation to maintain adherence [47]. The time and effort required to navigate multiple food support services impacted individuals’ ability to remember to take medication [45]. For those who accessed non-HIV specialist food support services, anxieties about revealing one's HIV status by taking ART publicly with food was a further barrier to adherence [45]. Finally, the pernicious effects of food insecurity on mental health (described later), especially anxieties about procuring food, had a deleterious effect on adherence [47].

The impact of food insecurity on HIV clinic attendance was relatively under-investigated; one study found that, overall, people living with HIV generally engaged well with HIV care despite multiple challenges [45]. However, for some, the physical experience of hunger and exhaustion sometimes impacted clinic attendance [45]. In one study with women experiencing interpersonal violence, the combined mental health impacts of food insecurity and violence led to missed clinic appointments [39].

#### Mental health

Food insecurity was described as a key contributing factor in stress, anxiety and depression among people living with HIV [45,39]. Not being able to afford enough food led to an “erosion of mental wellbeing” [47], accompanied by feelings of shame and failure, especially for those living in wealthier areas [45]. Food insecurity often exacerbated depression associated with living with HIV [45].

Access to food support can improve mental health, and increase energy, self-esteem and a sense of agency. One study sought to understand the experiences of participants enrolled on a food programme which provided three medically appropriate meals per day plus snacks [38]. The positive impact of this programme was multi-dimensional as it also reduced financial hardship more broadly [38]. Furthermore, by supporting others in their communities, individuals’ experienced improvements in their mental health [38].

### The pursuit of food

#### Strategies for acquiring food

Participants struggled to access healthy and good quality food [37]. Procuring sufficient food necessitated prioritizing quantity over quality, with participants relying on low quality “junk” food to avoid hunger [37].

People developed multiple strategies to obtain food, including stockpiling, relying on friends and family, and/or sharing resources with other people experiencing food insecurity [37]. Individuals also described checking in to homeless shelters (when housed) to obtain meals, selling prescription drugs, participating in research studies for money or vouchers, earning extra money without declaring to agencies, attending catered events, recycling bottles for money, stealing [37] and/or searching bins [37,43].

Three studies, with male and female participants often experiencing homelessness, described transactional sex to procure food [36,37,39]. This ranged from a long-term “livelihood strategy” taken as an active decision, to more of an impromptu “opportunistic encounter” [36]. For some women, transactional sex increased the risk of sexual violence, which was further exacerbated in contexts of drug use [39].

#### Experiences of food support services and programmes

Obtaining sufficient food often entailed accessing multiple food support services, with some participants describing doing “rounds” [44]. This required significant effort and knowledge of local services. Weekends and night-time were particularly challenging times to source food due to closure of support services [44].

Food from some food support services was described as low quality [37], of low nutritional value [44] and/or spoiled [44]. In one study, women described food support services as offering mainly starchy foods, with limited access to fruit and vegetables [41]. In another, conducted in Canada, all 10 participants had experienced food poisoning after accessing a food support service [44].

Some services employed a holistic model of care, which was valued by service users, offering laundry facilities and/or showers [44]. Food support services were described as sources of community, care and belonging [43,38,41] and as providing social opportunities[46]. For participants using drugs, they also offered a place of safety from the risks associated with drug use[43].

#### Barriers to accessing food support services and programmes

Six of the articles were conducted in areas with multiple food support services [36,37,41,43–45], including food banks, meal programmes, soup kitchens and community pantries. Four articles included participants enrolled on food programmes providing three meals a day [36–38,45].

Barriers to accessing food support services included travel costs [41], competing priorities such as family obligations [41], and restrictive opening times [41,37]. For those accessing specialist HIV food support services, some found having to share HIV status was a significant barrier [44,46].

Physical health was an important barrier, with limited mobility and ART side effects (e.g. fatigue) constraining individuals’ capacity to access services [41]. Additionally, participants described long queues [37], often at the end of the month when social support payments had run out [44]. These queues posed a significant impediment to access, especially for those who were unwell [44]. Furthermore, participants described arguments and fights [44], with queues sometimes perceived as physically unsafe [43]. This was exacerbated by the location of some food support services in areas considered to be unsafe [43]. For individuals who used drugs, the location of food support services in areas with high levels of drug use were also described as “triggering”[43].

Women had concerns about safety and/or the risk of sexual harassment in queues at food support services [41,44]. Such concerns were particularly acute for those with histories of trauma or violence [41]. Transgender women faced particular challenges in accessing food support, as women-only services may not be tailored to them or may make them feel unwelcome [44].

## Discussion

Our review highlights how food insecurity is shaped by structural inequities, and does not operate in isolation. People living with HIV and experiencing food insecurity face multiple material disadvantages, navigating competing financial demands, often in the context of unemployment or low wage work, insufficient state benefits, high rents and/or poor-quality housing. The mental health impacts of this, coupled with the labour required to obtain sufficient food, undermines ART adherence and engagement with care. As well as supporting nutrition, food support services offer social support and connection in the context of remarkably difficult circumstances. However, there are important barriers to accessing these services, often in relation to other experiences of marginalization.

Our findings lend support to qualitative research in LMICs [23–25,48] on the impacts of food insecurity on ART adherence. While our findings echo previous research in these settings, the experience of accessing food support and interacting with welfare systems is shaped by the specific structural conditions and institutional contexts of HICs. Additionally, although few included studies explicitly examined with the experiences of racially minoritized communities, it is likely that the particular forms of racism and social marginalization operating in HICs further shape how food insecurity is lived and managed. We also extend findings from a previous 2017 systematic review of interventions addressing food insecurity among people living with HIV in high income settings [27], which had limited engagement with the lived experiences of HIV and food insecurity.

Our review highlights a paucity of research on HIV and food insecurity in HICs, with no studies conducted outside of North America. This is an important gap to address, as findings from North America cannot be generalized to other settings, given differences in health and welfare systems. However, it is important to note that a large proportion of included studies were considered to be of a high quality, with diverse participant populations. Future research should be informed by this work and include a similar diversity of participants.

This review has significant clinical and policy implications. It highlights the importance of a non-stigmatizing, non-judgemental approach in clinical services, understanding that there may be structural barriers to implementing a healthy diet. HIV services should include food insecurity screening for patients, given the prevalence and impact. The findings also underline the importance of robust referral pathways between HIV clinical services and food support services. This review highlights the syndemic nature of food insecurity and HIV, where intersecting experiences of inequality and hardship combine and interact synergistically to amplify poor health outcomes. Interventions should therefore address the multiple and reinforcing social and structural conditions that shape food insecurity among people living with HIV. Importantly, those in the HIV sector should advocate for the particular needs of people living with HIV at a policy-level. Although some of the findings of this review may be applicable to other populations, people living with HIV are disproportionately vulnerable to changes in welfare provisions and the cost of living.

### Strengths and limitations of the review

We conducted a systematic search of the literature and standardized data extraction. Full text review, extraction, and quality and confidence assessment were conducted by two independent reviewers. A limitation is the exclusion of grey literature and studies not published in English. The lack of availability of studies outside a North American context limits transferability of findings. Finally, food insecurity is not defined, so definitions will vary across studies.

## Conclusion

Our review reveals the corrosive and syndemic impacts of HIV, food insecurity and other experiences of hardship and marginalization. As poverty and economic hardship are increasing in some HICs [49–51], and access to state benefits increasingly limited, growing numbers of people living with HIV will experience food insecurity. This has dire consequences on engagement in care and mental health. Our review highlights the importance of routine screening for food insecurity among people accessing HIV services (as we do for mental health and housing), and providing services that address multiple material needs.

## Acknowledgements

Funding: This work was partially funded by a Central and North West London NHS Foundation Trust Research Starter Grant.

This work was partially funded by the NIHR Health Research Unit in Blood Borne and Sexually Transmitted Infections at University College London (NIHR200911), in partnership with the UK Health Security Agency (UKHSA). We acknowledge members of the National Institute for Health and Care Research Health Protection Research Unit (NIHR HPRU) in Blood Borne and Sexually Transmitted Infections (BBSTI) Steering Committee: Professor Caroline Sabin (HPRU Director), Dr John Saunders (UKHSA Lead), Professor Catherine Mercer, Professor Gwenda Hughes, Dr Hamish Mohammed, Professor Greta Rait, Dr Ruth Simmons, Professor William Rosenberg, Dr Tamyo Mbisa, Professor Rosalind Raine, Dr Sema Mandal, Dr Rosamund Yu, Dr Samreen Ijaz, Dr Fabiana Lorencatto, Dr Rachel Hunter, Dr Kirsty Foster and Dr Mamooma Tahir.

The views expressed in this article are those of the author(s) and not necessarily those of UCL, UKHSA, the NIHR or the Department of Health and Social Care.

This article is available as a preprint on SSRN: http://dx.doi.org/10.2139/ssrn.5844582.

### Conflicts of interest

None of the authors have conflicts of interest to declare.

## Supplementary Material

Supplemental Digital Content
