# Supplementary material for: A systematic review of qualitative research on HIV and food insecurity in high-income countries
Source: AIDS. 2026 May 25;40(10):1539–51. doi: 10.1097/QAD.0000000000004552 (PMC13566418; doi:10.1097/QAD.0000000000004552)
Supplement: Supplementary file 1 [file aids-40-1539-s001.docx]

**Search strategy**

HIV OR human immunodeficiency virus OR human immune deficiency virus OR acquired immunodeficiency OR PLWH* OR antiretroviral*

AND

food insecur* OR food secur* OR food scarc* OR food poverty OR food short* OR food stamp* OR food bank* OR malnourish* OR undernourish*

AND

qualitative OR Interview* OR focus group* OR ethnograph* OR case stud* OR lived experience* OR thematic OR diar* OR visual methods OR photovoice

**Databases and field tags**

**Medline (Ovid)**

ab=abstract

kf=keyword heading word

ti=title

**CINAHL Plus (EBSCOhost)**

Xb = abstract, title

**Scopus**

TITLE-ABS-KEY= title, abstract, keywords

**EMBASE (Ovid)**

ab=abstract

kf=keyword heading word

ti=title

**PsycINFO (Ovid)**

ab=abstract

kf=keyword heading word

ti=title
